# Are Face Masks a Problem for Emotion Recognition? Not When the Whole Body Is Visible

**DOI:** 10.3389/fnins.2022.915927

**Published:** 2022-07-18

**Authors:** Paddy Ross, Emily George

**Affiliations:** Department of Psychology, Durham University, Durham, United Kingdom

**Keywords:** COVID-19, face masks, emotion recognition, body perception, face emotion

## Abstract

The rise of the novel COVID-19 virus has made face masks commonplace items around the globe. Recent research found that face masks significantly impair emotion recognition on isolated faces. However, faces are rarely seen in isolation and the body is also a key cue for emotional portrayal. Here, therefore, we investigated the impact of face masks on emotion recognition when surveying the full body. Stimuli expressing anger, happiness, sadness, and fear were selected from the BEAST stimuli set. Masks were added to these images and participants were asked to recognize the emotion and give a confidence level for that decision for both the masked and unmasked stimuli. We found that, contrary to some work viewing faces in isolation, emotion recognition was generally not impaired by face masks when the whole body is present. We did, however, find that when viewing masked faces, only the recognition of happiness significantly decreased when the whole body was present. In contrast to actual performance, confidence levels were found to decline during the Mask condition across all emotional conditions. This research suggests that the impact of masks on emotion recognition may not be as pronounced as previously thought, as long as the whole body is also visible.

## Introduction

Accurate emotion recognition enables humans to sustain relationships crucial for survival, cooperative living, and reproduction (Chen, 2019). Most non-verbal emotion recognition chiefly relies on facial and bodily cues (de Gelder and Van den Stock, 2011; Jack and Schyns, 2015) and successful non-verbal interactions can only occur if there is mutual understanding and recognition of what the other party is expressing.

The face is arguably the most informative visual stimulus in human perception (Ekman et al., 1980). The face’s high social visibility, accessibility and expressiveness make it a prime vehicle for exchanging emotional information (Hager and Ekman, 1979; Schmidt and Cohn, 2001). However, facial emotion recognition can be impaired by facial occlusion (Wegrzyn et al., 2017). Indeed, when a person’s face is obscured, the perceiver’s emotion recognition ability is impaired, as they are left with only the remaining visible social information to rely on (Spitzer, 2020). Therefore, they tend to reconstruct the face to make an informed interpretation—with the mouth and eyes having more weight on correct reconstruction (Nestor et al., 2020).

How distinct types of occlusions interfere with emotion recognition is contingent on the emotions themselves. There has been culturally varied research on the impact of partial occlusions, such as from shawls, caps (Kret and De Gelder, 2012) and niqabs (Fischer et al., 2012). It was found that when only the upper half of the face was visible, participants were able to accurately recognize negative emotions more readily than positive ones—thus suggesting the eyes are more integral for expressing more negative emotions. For example, Nestor et al. (2020) argue that the squinting of the eyes from smiling can be misconstrued as skepticism, therefore indicating occlusion of the mouth may enhance the perception of negative emotions while reducing positive perceptions. Indeed, Maurer et al. (2002) argue that partially obscuring a face disrupts holistic face processing, meaning certain facial features cannot be processed relationally to other features. For example, if a person were wearing a niqab, the perceiver would be unable to create a coherent gestalt of the face, as their processing of the eyes in the context of having a nose and a mouth beneath it would be disrupted.

In the current climate of the COVID-19 pandemic—faces are regularly partially obscured by face masks. Whilst masks help prevent the spread of viruses and protect those most at risk (Wu and McGoogan, 2020), they also propel humanity into a unique conundrum when trying to read a masked individual’s non-verbal emotional expressions (Pavlova and Sokolov, 2022). Because masks cover ∼ 60–70% of the face relevant for emotional expression (Carbon, 2020), observers only have the top half of the face for emotion detection. Nestor et al. (2020) argued this new, masked norm brings with it a potentially “radical change to human psychosocial dynamics and communication.” Indeed, whilst previous research indicates that occlusion of the mouth area has a significant impact on accurate recognition of happy states, up until the beginning of the Covid-19 pandemic, there was limited research regarding the actual implications of mask- wearing (Chua et al., 2020).

That being said, Carbon (2020) study proposes a link between face-mask wearing and decreasing ability to recognize an individual’s emotion. Whilst finding a clear performance drop in emotion recognition accuracy viewing masked stimuli (except for fearful and neutral faces), they also observed a marked drop in confidence levels during the mask condition.

There have been several other recent papers describing decreases in emotion recognition in faces (Ruba and Pollak, 2020; Grundmann et al., 2021; McCrackin et al., 2022), identity and expression recognition (Carragher and Hancock, 2020; Noyes et al., 2021; Grahlow et al., 2022; Kim et al., 2022), expression intensity (Tsantani et al., 2022), memory for faces (Freud et al., 2020), connectedness to the speaker (Mheidly et al., 2020; Saunders et al., 2021), perception of attractiveness (Parada-Fernández et al., 2022) and trust attribution (Biermann et al., 2021; Marini et al., 2021). Face masks would therefore appear to have quite a detrimental effect on normal social interaction. All of these studies, however, have something in common; they use stimuli in which the face is the only body part visible.

However, in situations where one might see a face mask (during face-to-face interactions rather than online interactions) faces are rarely seen in isolation. Facial expressions are nearly always seen as an indistinguishable entity of social information, accompanied by body language, vocal cues, hand gestures and posture. Indeed, emotions can be reliably recognized from the body alone when facial information is removed (Atkinson et al., 2004; Van den Stock et al., 2007; Ross et al., 2012; Ross and Flack, 2020), suggesting that the body and the face yield similar emotion detection capacity.

In the first comprehensive review on reading masked faces, Pavlova and Sokolov (2022) argue that while facial expressions may be kept under reasonable control, body movements can reveal our true feelings. As such, they argue that given the rich sources of non-verbal information such as body language and prosody available to a viewer, these should also be taken into account when investigating the impact of face masks on social interaction. This is something which in our opinion has not yet been explored in any detail in the literature.

Previous literature also argues that emotional face recognition is significantly improved when in conjunction with congruent bodily expressions (Kret et al., 2013). Mondloch (2012) further contends that when there is a disruption to this congruence, such as a sad face paired with an angry body, individuals will preferentially attend to the face over the body to make an emotional recognition decision. Therefore, if a mask if partially obscuring a face, this could affect the integration of face-body emotional information resulting in ambiguous or unclear emotion recognition. This is reflected in eye-tracking data, which reveals that during social interactions, there are more frequent and more prolonged fixations on the face compared to the body (Shields et al., 2012). Whilst this is true during general interaction, research suggests eye movements fluctuate depending on the emotion portrayed (Kret et al., 2013). For example, eye movements attended bodily cues (particularly the hands) to a greater extent when perceiving angry and fearful emotions than happy or sad expressions (Fridin et al., 2009). In most “body-only” stimuli the face is blurred out, forcing participants to determine the emotion from the body. It could be the case therefore, that in situations where the face is obscured with a mask, the body “picks up the slack” and identification is still possible. Alternatively, it could be the case that as above, when the face is present (masked or not), it is the preferred modality for emotion recognition, and the presence of the body has little impact on recognition accuracy. Will the findings of a detrimental impact of masks (which one can see throughout the literature) hold when the body is available?

Although there has been work researching the impact of face masks using the face in isolation, to our knowledge, this is the first exploration of the impact of face masks whilst surveying the entire expressive body. Therefore, unlike other studies using isolated faces, this study will feature the whole body in conjunction with the face, thus creating a more realistic presentation of encountering a mask-wearing individual. Using a full-body set of static stimuli (de Gelder and Van den Stock, 2011), this study investigates whether wearing a face mask impacts emotion recognition accuracy when surveying the full-body for anger, happiness, sadness, and fear. To further delve into this concept, similar to Carbon (2020), the individual’s reported confidence levels when making that initial deduction of emotion recognition were also investigated.

It was first hypothesized that face masks would significantly impair the general accuracy of emotion recognition for all emotion conditions except fear, reflecting Carbon (2020) study. We further hypothesized that reports of confidence in the accuracy of their choice would be significantly lower in all emotions with a mask than without a mask.

## Methodology

### Participants

We conducted a power analysis to justify our choice of sample size. We based our predicted effect size on calculations in the surgical face mask paper of Carragher and Hancock (2020). They in turn took the effect size from Kramer and Ritchie (2016) which was η^2^ = 0.13 in a one-way ANOVA with three conditions. An a priori power analysis indicated that a total of 62 participants would be needed to achieve 80% power to detect an effect of η^2^*_*p*_* = 0.15 in a 2 × 4 within subjects ANOVA as we have here. Therefore 70 participants from the United Kingdom (48 females, 21 males, 1 non-binary; mean age = 29.5, *SD* = 11.4) volunteered and were recruited to participant. All participants provided informed consent and were recruited through the Durham University Psychology Participant Pool and on Prolific Academic. Psychology students completed the experiment in exchange for course credits and prolific academic participants were renumerated for their time.

### Stimuli and Materials

**FIGURE 1:**
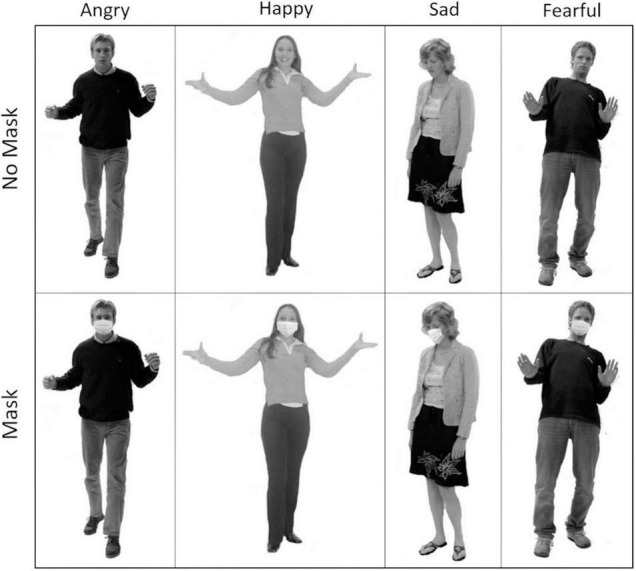
Examples of the manipulated BEAST stimuli displaying the 4 different emotions in the Mask **(top)** and No Mask **(bottom)** conditions. The emotions from left to right; Anger, Happiness, Sadness, Fear.

Stimuli were taken from the Bodily Expressive Action Stimuli Test (BEAST) (de Gelder and Van den Stock, 2011), which consists of 254 posed whole-body images of 46 actors expressing four emotions (Happiness, Sadness, Anger and Fear). Eighty stimuli (20 for each emotion, 10 males and 10 females) were selected from the total set. Manipulations of the BEAST images were then completed using the photo editing software GNU Image Manipulating Program (GIMP) to create the Mask conditions. Stock images of masks were found from Google Images. These images were individually manipulated to fit onto different stimuli’s faces. As in Carragher and Hancock (2020) masks were fitted over the stimuli such that they covered the same features of the face as is recommended in real life; i.e., covering the face from the middle of the nose to below the chin. We also made sure that when the head is slightly turned the mask is seen coming under the chin. Mask straps and shadows were also added to maximize ecological validity. Examples of stimuli can be seen in Figure 1.

### Design and Procedure

The experiment was conducted using the online survey platform Qualtrics. Each participant saw all 160 stimuli in a randomized order. The stimuli were presented for 4 s. After that, participants were presented with a forced-choice of which emotion they believed the stimuli were expressing—Anger, Happiness, Sadness, or Fear. This was presented in the form of a multiple-choice button. Participants saw both masked and unmasked stimuli and were then asked to assess their confidence level in the accuracy of this choice by using a sliding bar from 0 (Not Confident) to 100 (Extremely Confident). Once this was completed, the participants clicked the “Next” button, and the procedure was repeated with the subsequent randomized stimuli and question set. The procedure lasted approximately 30 min.

## Results

### Accuracy for Emotion Recognition

Percentage correct emotion identification scores were calculated for each emotion/condition combination for each participant and averaged across all participants to give an overall percentage correct response measure. This gave us a 2 (Mask condition) × 4 (Emotion) design. However, upon inspection of the data distributions using histograms and Q-Q plots it was decided that they did not look normally distributed. A Shapiro-Wilk test confirmed this (*W* = 0.839, *p* < 0.001) and thus non-parametric analyses were performed.

**FIGURE 2:**
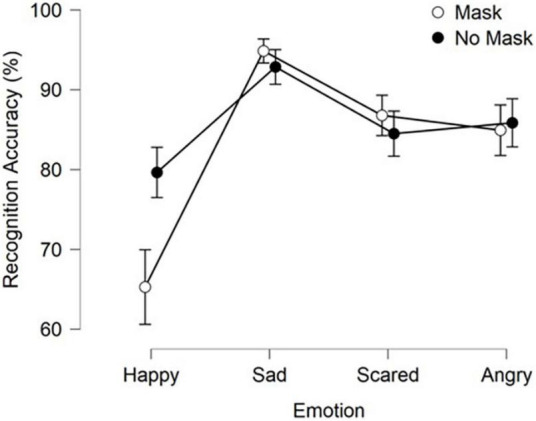
Average recognition rates for both Mask conditions and the four emotions. Error bars represent 95% confidence intervals.

Performing separate Friedman Tests for each factor we first found a significant main effect of Emotion type on recognition ability X^2^ (3) = 152.07, *p* < 0.001, Kendall’s *W* = 0.36. Conover’s *post-hoc* comparisons and applying Bonferroni correction revealed that Sadness was recognized significantly more accurately than Happiness (*p* < 0.001), Fear (*p* = 0.008) and Anger (*p* = 0.01) (see Figure 2).

Friedman Tests for the Mask condition, however, revealed no significant main effect on emotion recognition ability X^2^ (1) = 0.34, *p* = 0.561, Kendall’s W = 0.012.

With no clear way of checking for an interaction effect on 2 × 4 non-parametric repeated-measure data, we instead ran 4 Wilcoxon signed-rank tests looking at the differences between the two Mask conditions for each of the 4 Emotion types. Using Bonferroni corrections we found that Happiness was significantly harder (*T* = 83, *z* = –6.27, *p* < 0.001) to recognize in the Masked condition (Median = 70%) compared to the No Mask condition (Median = 80%). We found no significant difference between the Mask conditions across the other 3 emotion types (Sadness: *T* = 805, *z* = 2.23, *p* = *n.s*; Fear: *T* = 871.5, *z* = 2.26, *p* = *n.s*.; Anger: *T* = 654, *z* = –0.54, *p* = *n.s*.).

### Confusion Matrices

**FIGURE 3:**
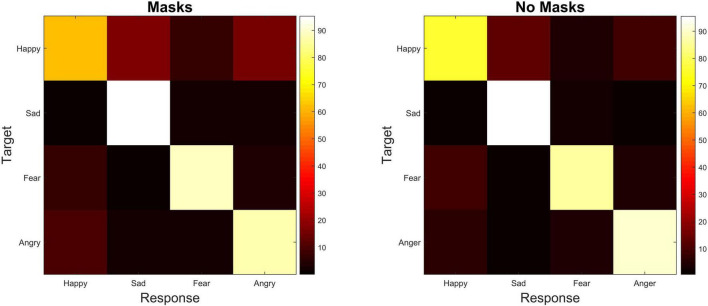
Confusion matrices showing correct emotion response to target emotion (Happiness, Sadness, Fear and Anger) in the two masked conditions (Mask and No Mask). The color bar represents % of responses.

We know that between the two mask conditions, Happiness was found to be significantly harder to recognize when the face was masked (*T* = 83, *z* = –6.27, *p* < 0.001). Here we wanted to explore whether this effect was due to Happiness being confused for one other emotion specifically. The confusion matrices indicate that within our Mask condition Happiness was not being confused with one emotion in particular, but rather it was confused for Sadness 13.9%, Angry 13.4% and Fear 7.4% of the time (see Figure 3). This means that the particularly low emotion recognition rate of Happiness in the Mask condition wasn’t because it was being confused with any other emotion in particular, rather there was a spread of incorrect responses across the other 3 choices.

### Confidence Levels for Emotion Recognition

**FIGURE 4:**
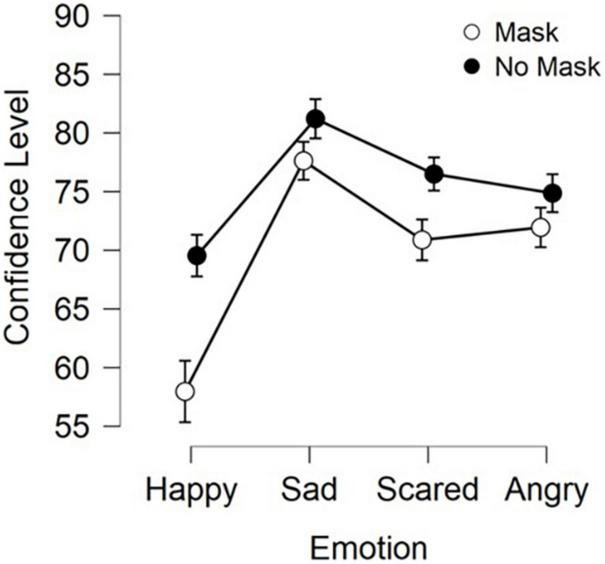
Average confidence levels for both Mask conditions and the four emotions. Error bars represent 95% confidence intervals.

As with emotion recognition, the average confidence scores were recorded and entered into a 2 (Masked condition) × 4 (Emotion) analysis of variance (ANOVA). See Figure 4 for summary of results. Upon inspection of the data distributions using histograms and Q-Q plots it was decided that these data did look normally distributed. This was confirmed by a Shapiro-Wilk test (*W* = 0.989, *p* = 0.783), and thus repeated measure ANOVA were performed.

We found a main effect of emotion [*F*(3, 207) = 69.23, *p* < 0.001]. Bonferroni corrected post-hoc tests showed this to be driven by participants being significantly less confident in their answers for Happiness compared to Sadness (*t* = 14.19, *p* < 0.001), Fear (*t* = 9.0, *p* < 0.001) and Anger (*t* = 8.75, *p* < 0.001) as well as showing more confidence in answers for Sadness than Fear (*t* = 5.19, *p* < 0.001) and Anger (*t* = 5.44, *p* < 0.001).

We also found a main effect of Mask [*F*(1, 69) = 60.4, *p* < 0.001] with confidence in recognition of Masked stimuli (Mean = 69.6%) being significantly lower than No Mask stimuli (Mean = 75.53%).

Finally we found a significant interaction between Masked Condition and Emotion [*F*(3, 207) = 26.73, *p* < 0.001]. Contrary to our recognition accuracy data, Bonferroni corrected paired *t*-tests showed that this interaction was driven by lower confidence in the Masked condition compared to the No Mask condition in all emotions (Happiness: *t* = 11.5, *p* < 0.001; Sadness: *t* = 3.56, *p* < 0.005; Fear: *t* = 5.57, *p* < 0.001; Anger: *t* = 2.89, *p* < 0.05).

## Discussion

The present study aimed to explore whether face masks impaired emotion recognition when surveying the full body. We first hypothesized that emotion recognition accuracy would be impaired in all emotions except fear, mirroring Carbon (2020) study. Secondly, we predicted that confidence would be lower when determining the emotion in masked individuals. Our results partially support these hypotheses.

In terms of the first hypothesis, and contrary to the work using isolated faces by Carbon (2020), we instead found that emotion recognition accuracy was only affected when masked full-body stimuli were portraying happiness. We found no detrimental effects of masks in the recognition of sadness, fear or anger.

Despite this largely unaffected recognition, we found that confidence levels in responses were lower for the Masked condition across all emotions.

### Emotion Recognition

These results suggest that when the face is not seen in isolation, the impact of mask-wearing on emotion recognition ability is largely unchanged. The exception to this is Happiness, which is primarily portrayed using the face, and the recognition of which in the body has been shown to be ambiguous at times (de Gelder and Van den Stock, 2011; Ross et al., 2012; Ross and Flack, 2020).

Even when unmasked, the drop off in recognition rates for happiness is reflected in several previous studies surrounding emotion recognition from the body (Atkinson et al., 2004; de Gelder and Van den Stock, 2011; Ross and Flack, 2020). This is also consistent with research that contests that happiness is often expressed through the mouth with a smile (Calvo and Nummenmaa, 2016). Indeed, Gavrilescu (2017) found the addition of hand and bodily cues did not substantially increase the recognition of happiness compared to the addition of happy facial expressions. Therefore, this suggests that recognition of happy states is more heavily reliant on the face than the body. In addition to crucial facial information being covered, and as previously mentioned, past research indicates that happy bodily cues can also be ambiguous (de Gelder and Van den Stock, 2011; Ross et al., 2012; Witkower and Tracy, 2019; Ross and Flack, 2020), with Tracy and Robins (2007) finding happy bodily cues are often misinterpreted as other emotions, such as neutrality and pride. If a body is ambiguous and we are unsure, we then may turn to the face for confirmation. If that face then has the main cue for determining happiness obscured, this would perhaps explain the effect that masks have on recognition rates for Happiness. Indeed, we see in our confusion matrices that, on average, participants are confusing Happiness with Sadness and Anger 13.9 and 13.4% of the time, respectively, when people are masked. This may indicate that instead of looking like another emotion in particular, participants are guessing in these instances. Alternatively, these stimuli could look like slightly different emotions for which there is no option in the current design (e.g., exasperation). We have suggested possible solutions to this later in the discussion.

However, contrary to happiness, the lack of impairment in the other emotions is in stark contrast to Carbon (2020) study, which found a significant discrepancy in accuracy recognition when facial stimuli were masked. These emotions arguably have distinctive body postures and configurations which differentiate them (Dael et al., 2012). The slumped shoulders and bent neck are uniquely indicative of sadness, while raised hands as shields or fists construe fear or anger, respectively, (Coulson, 2004; Rosario et al., 2014). Therefore, even with the face obstructed with a mask, the head positioning and body posture is still salient, explaining its easy recognition.

It is also true that fear and anger are most effectively expressed through the eyes and brow – the area left visible by masks (Gosselin and Schyns, 2001; Fischer et al., 2012; Wegrzyn et al., 2017). Alongside anger, fear has particularly expressive body language, as such threat-based expressions are often highly animated as they precede evolutionary actions such as fleeing or fighting (Martinez et al., 2016). In these static stimuli, hand positioning is especially distinctive during angry and fearful emotional states (Ross and Flack, 2020), with common fearful reactions including open palms protecting one’s face (Grèzes et al., 2007). In fact, eye-tracking indicates that individuals look longer at the hands of angry and scared stimuli when making emotional judgments (Fridin et al., 2009). This suggests that with the faces masked, either individuals rely more on the remaining bodily information, or that the face is simply not needed to recognize emotion when the whole body is present. Either way, masks do not hinder emotion recognition rates in these stimuli.

### Confidence Levels

Despite recognition accuracy remaining unchanged for sadness, fear and anger, confidence levels in responses across all emotions significantly reduced when participants were observing masked stimuli. This is reflected in Carbon (2020) paper, in which he also found a significant reduction in confidence. Such confidence reductions are aligned with the aforementioned models regarding holistic face processing (Maurer et al., 2002). Presumably, the simplest explanation probably holds here, that by presenting less information to people, confidence in one’s ability to recognize some aspect of that person will be lowered.

A possible explanation for this reduction in confidence yet intact emotion recognition ability could be the presence of implicit bias. In other words, the assumption that masks will inevitably impact their ability to perceive emotions diminishes their confidence levels. However, the recognition accuracy results indicate that this is not necessarily correct in practice. This pattern of low reported confidence yet intact competence is reflected in Lorey et al. (2012) and their research with alexithymia individuals. A possible implication of this finding is that reduced confidence could result in lowered willingness to engage in pro-social behavior, for fear of misjudging emotional interactions, even though perception remains largely intact.

### Implications and Limitations

Here, we have presented evidence that, contrary to previous work investigating the effect of face masks on emotion recognition, when presented with whole bodies rather than isolated faces emotion recognition remains largely unaffected. This should allay some concerns raised by previous work regarding the problems masks raise in emotion recognition. It further implies that when wearing a mask, by emphasizing an emotion through body language, there should be no notable reduction in recognition. This is particularly pertinent for happiness, which did show a significant decrease in recognition accuracy. One could imagine a situation where if our body stimuli contained a distinct “happy” hand signal (e.g., thumbs up) we may have seen no reduction in recognition accuracy.

One limitation of the current study, however, is that although being arguably more ecologically valid than face-only stimuli, the results are still quite specific to these particular stimuli. Here actors were asked to imagine a scenario and act it out (then a photo was taken). For happiness they were asked to imagine seeing a long-lost friend at a train station. Thus, our happy body stimuli have their arms outstretched and are arguably more ambiguous compared to the others.

This leads to another issue with these particular stimuli, namely the lack of nuance in the emotions presented. By adding emotions that are less apparent from the body (embarrassment or pride), this may “force” participants to look at the face. Indeed, including a condition in which the faces of the stimuli are seen in isolation would be of great benefit. Here, the resolution of the current stimuli would not allow us to explore this effectively, but by including a face only condition, the contribution of the face in these stimuli compared to the body could be examine in more depth. Although it is not our main intention here, investigating the extent to which the face is “used” in emotion recognition when the face is masked and the whole body is visible would be a very interesting avenue of research to pursue. Either through eye-tracking or fMRI one could imagine quantifying the amount of attention the face gets in these stimuli and understanding whether the body is the main focus.

One must also consider the potential gender differences across participants as here we have a 2:1 gender split in favor of females. Although we find no differences in recognition rates across males and females in the current study, previous research has found that females tend to score higher in emotion recognition tasks at a variety of ages (Grosbras et al., 2018; Abbruzzese et al., 2019; Olderbak et al., 2019). Future work should bear this in mind and endeavor to achieve an equal gender split in participants.

Age as well may play a role in our results. Looking at our confusion matrices, a possible explanation for Happiness being confused more than other emotions could be the positive bias in emotion perception described in the literature for older individuals/negative bias in younger individuals (Di Domenico et al., 2015). Our participants were on average 29 years old, so perhaps Happiness was being recognized as one of the other 3 negative emotions in this case more often than perhaps it might have been had our participants been older. One way to counter this possibility in the future is to have a balance of “positive” and “negative” emotions, instead of the 1:3 ratio we have here.

Future work may also want to remove the forced choice element of the design. Happiness in the Mask condition was mostly confused with Sadness and Anger, but in a forced choice task the confusion is also forced. Perhaps the stimuli actually looked confused, or showed annoyance, and the labels provided were the closest available alternative. By allowing open choice emotional questioning it would allow for more nuance to be observed in the stimuli, and the effect of masks and emotion recognition to be explored with more emphasis on the potential confusion and ambiguity created by masks.

In our view using whole bodies in this context is a more ecologically valid method than using isolated faces. However, they are static images which are still suboptimal. Dynamic videos would be preferable, with research showing that emotion recognition is considerably improved when observing moving stimuli (Dittrich et al., 1996; Atkinson et al., 2004; Dael et al., 2012). Perhaps stimuli with less extreme poses of the given emotions would be preferable, either taken from media (e.g., films) or created specifically for this purpose.

## Conclusion

To conclude, this study reveals novel results with globally reaching implications. It uncovers the first evidence of emotion recognition from the full body whilst wearing masks and demonstrates that aside from happiness, recognition is unaffected. It also serves to allay some of the concern previously raised by research suggesting that masks severely impact emotion recognition. Indeed, such studies surrounding the COVID-19 pandemic have suggested that the rise of face masks may bring with it a new dawn of compromised emotional communication (Carbon, 2020; Nestor et al., 2020). However, whilst confidence levels generally decline and emotion recognition of happiness decreases when these stimuli are masked, using these stimuli recognition for other emotions is left unchanged. This suggests that the impairment is perhaps not severe enough to warrant any considerable implications for most emotional interactions. It does suggest that we could express happiness in different ways, particularly regarding making more overt gestural expressions of happiness (Mheidly et al., 2020). This is especially important when one considers the impact that face masks have had on those with hearing loss. In a recent study, over 80% of adults who were deaf or hard of hearing reported difficulty understanding others who wore face masks (Poon and Jenstad, 2022). Therefore, it should be noted that these types of studies focusing on visual posed stimuli do so while negating the vocal modality. In a social interaction one is likely to be able to hear the other person as well as view their whole body, thus future work could also incorporate vocal emotions and explore multimodal emotion recognition with dynamic stimuli to further increase ecological validity.

## Data Availability Statement

The datasets presented in this study can be found in online repositories. The names of the repository/repositories and accession number(s) can be found below: https://osf.io/p5eh4/.

## Ethics Statement

The study was approved by the Durham University Psychology Department ethics committee, reference: PSYCH-2020-07-02T21_48_20-dwqg21. The patients/participants provided their written informed consent to participate in this study. Written informed consent was obtained from the individual(s) for the publication of any potentially identifiable images or data included in this article.

## Author Contributions

PR and EG designed, conducted the study, and drafted the manuscript. EG created the stimuli. PR analyzed the data. Both authors read and approved the final manuscript.

## Conflict of Interest

The authors declare that the research was conducted in the absence of any commercial or financial relationships that could be construed as a potential conflict of interest.

## Publisher’s Note

All claims expressed in this article are solely those of the authors and do not necessarily represent those of their affiliated organizations, or those of the publisher, the editors and the reviewers. Any product that may be evaluated in this article, or claim that may be made by its manufacturer, is not guaranteed or endorsed by the publisher.
